# Band Gap Energy and Lattice Distortion in Anatase TiO_2_ Thin Films Prepared by Reactive Sputtering with Different Thicknesses

**DOI:** 10.3390/ma18102346

**Published:** 2025-05-18

**Authors:** Cecilia Guillén

**Affiliations:** Centro de Investigaciones Energéticas Medioambientales y Tecnológicas (CIEMAT), Avenida Complutense 40, 28040 Madrid, Spain; c.guillen@ciemat.es

**Keywords:** semiconductors, thin films, optical properties

## Abstract

TiO_2_ is an abundant material on Earth, essential for the sustainable and cost-effective development of various technologies, with anatase being the most effective polymorph for photocatalytic and photovoltaic applications. Bulk crystalline anatase TiO_2_ exhibits a band gap energy E_gA_ = 3.2 eV, for tetragonal lattice parameters a_A_ = 0.3785 nm and c_A_ = 0.9514 nm, but these characteristics vary for amorphous or polycrystalline thin films. Reactive magnetron sputtering has proven suitable for the preparation of TiO_2_ coatings on glass fiber substrates, with structural and optical characteristics that change during growth. Below a minimum thickness (t < 0.2 μm), the films have an amorphous nature or extremely small crystallite sizes not observable by X-ray diffraction. Afterwards, compressed quasi-randomly orientated crystallites are detected (volume strain ΔV = −0.02 and stress σ_V_ = −3.5 GPa for t = 0.2 μm) that evolve into relaxed and preferentially (004) orientated crystallites, reaching the standard anatase values at t ~ 1.4 μm with σ_V_ = 0.0 GPa. The band gap energy increases with lattice distortion according to the relation ∆E_g_ (eV) = −6∆V, and a further increase is observed for the thinnest coatings (∆E_g_ = 0.24 eV for t = 0.05 μm).

## 1. Introduction

Titanium dioxide (TiO_2_) is an abundant material on Earth, destined to play a pivotal role for the sustainable and cost-effective development of several technologies, including photocatalytic hydrogen production [1], photocatalytic pollutant removal [2], and photovoltaic power generation [3]. Titanium dioxide has various polymorphic forms, of which amorphous, rutile, and anatase are the most common [3]. While the rutile phase can play an important role in several applications [4], especially when mixed with anatase in modified TiO_2_ nanostructures [5], anatase is considered the most effective for photocatalysis and photovoltaics. This is due to its higher conduction band edge, which increases the reduction potential for electron transfer reactions, and its lower electron-hole recombination rate, which improves the lifetime of photogenerated charge carriers. Furthermore, anatase is generally formed into smaller grains with a larger surface area, which increases the number of active sites for photocatalytic reactions. In particular, the {001} facets of anatase are highly reactive and can further increase its photocatalytic performance [6,7].

For further development, it is crucial to produce TiO_2_ using techniques with high deposition rates and easy scale-up to large areas. Reactive magnetron sputtering meets these requirements and is suitable for the preparation of TiO_2_ thin films on different substrates [8,9]. It offers additional benefits such as optimal surface coverage and minimal material waste [10]. Both anatase and rutile TiO_2_ can be obtained by reactive sputtering depending on the total energy flux [11], which can be controlled by deposition parameters such as the discharge voltage [12] or the working gas pressure [13]. The formation of pure anatase requires a lower energy flow than the rutile phase, and it is common to obtain a mixture of both phases in varied proportions.

Since the properties of anatase and rutile phases are quite different, their identification as pure phases and the determination of the proportion of each of them in a mixture are important for understanding the performance of real devices [14]. Therefore, several methods have been developed to identify them and calculate their relative amounts, usually by X-ray diffraction (XRD) [14] or optical reflectance measurements [15]. The first method is based on the different lattice parameters for perfect anatase (a_A_ = 0.3785 nm, c_A_ = 0.9514 nm) and rutile (a_R_ = 0.458 nm, c_R_ = 0.295 nm), taking the ratio between the intensities of the anatase (101) and rutile (110) diffraction peaks to evaluate the mixtures [14]. Alternatively, the optical method is based on the different band gap energy for bulk anatase (E_gA_ = 3.2 eV) and rutile (E_gR_ = 3.0 eV), fitting the derivative of the diffuse reflectance spectra to Gaussian functions centered at the E_gA_ and E_gR_ values for the mixtures [15]. These considerations apply directly to bulk crystalline TiO_2_ samples, but a more detailed analysis is needed in the case of amorphous thin films or coatings with crystalline defects that alter both the lattice parameters and the E_g_ value even for single-phase samples [16].

This paper looks into the relationship between the structural and optical characteristics of TiO_2_ thin films deposited by reactive magnetron sputtering on unheated glass fiber wafers. These opaque glass fiber wafers are commonly used to support powdered photocatalytic materials because they facilitate gas or water filtration compared to flat glass substrates, and they are also less heavy and fragile. However, transparent flat glasses are more widely used for supporting thin films because they allow optical transmittance measurements. Here, the optical characterization is based on total and diffuse reflectance spectra obtained in a spectrophotometer equipped with an integrating sphere, while the structural characterization has been performed using XRD measurements. It is known that the band gap energy and lattice parameters can vary with the TiO_2_ film thickness, especially when it is less than 1 μm [13], and the current objective is to determine their evolution during sputtering growth on glass fiber wafers. The ultimate goal is to improve our understanding of the thin film material by relating its essential structural and optical characteristics, in order to achieve general expressions that are independent of the substrate or the preparation method used. The experimental results presented below show that structural deformation control can be a powerful tool for the improvement of photocatalytic and photovoltaic devices based on anatase TiO_2_ thin films, by allowing the better matching of their band gap energy to the illumination source.

## 2. Materials and Methods

The TiO_2_ coatings were prepared by the reactive DC magnetron sputtering of a metal target (15 cm diameter, 99.6% pure Ti disk) on unheated glass fiber wafers (13 cm diameter), which were placed 10 cm in front of the target. The deposition chamber was evacuated by means of a turbomolecular pump to a base pressure of 3 × 10^−4^ Pa and then boosted to a higher working pressure by introducing reactive (O_2_) and inert (Ar) gases through separate mass flow controllers. The partial pressures were set to P(O_2_) = 0.08 Pa and P(Ar) = 0.32 Pa, which were optimized in a previous work to obtain anatase TiO_2_ layers at a deposition rate of 10 nm/min on flat glass substrates [13]. The sputtering power density was kept constant at 8 W/cm^2^, and the sputtering time was varied to obtain different film thicknesses from 0.05 to 1.4 μm on the glass fiber wafers, where the ratio of surface area to geometric area is 35% higher than for flat glass substrates [10]. In the previous experimental study, simultaneous growth on both substrate types (fiber wafer and flat glass) was compared, and the same features were observed at the corresponding thickness [10], which is lower on the fiber wafer because the same amount of incoming TiO_2_ per unit time covers a larger surface area. The objective here is to better analyze the influence of the growth thickness on the structural and optical characteristics of anatase TiO_2_ thin films.

The microstructure of the samples was examined using a B&WTek system consisting of a BAC151B microscope (with a ×100 objective) and an i-Raman spectrometer (with a green laser of 532 nm) (both from B&W Tek, Newark, NJ, USA). The crystallographic structure was analyzed in a Philips X’pert diffractometer (PANalytical, Malvern, UK) with a Bragg–Brentano θ–2θ configuration and Cu Kα1 (λ = 1.54056 Å) radiation. The angular positions of the measured diffraction peaks were used to identify the crystalline phase by comparison with the standard Powder Diffraction Files (PDFs), while the full width at half-maximum of the diffraction peak allowed the calculation of the mean crystallite size [17]. The optical properties were determined from reflectance measurements with a Lambda 9 spectrophotometer (PerkinElmer Inc., Waltham, MA, USA) containing an integrating sphere coated on the inside with a BaSO_4_ diffuse reflectance standard. The determination of the total, diffuse, and direct (or specular) reflectance requires three scans as follows [18]: (1) a reference scanning without the sample, with BaSO_4_ plates covering the sample holder and the specular exit port, to set 100% light reflection from the reflectance standard; (2) a total signal scanning, with the material to be measured in the sample holder and a BaSO_4_ plate covering the specular exit port; and (3) a diffuse signal scanning, with the sample in its holder and nothing in the specular exit port. In this last configuration, only the scattered part of the radiation contributes to the detector signal because the specular beam is lost through the corresponding exit port. These readings allow us to obtain the total reflectance (R_tot_), the diffuse reflectance (R_diff_), and then the direct reflectance as R_dir_ = R_tot_ − R_diff_. Moreover, the absorptance is A(%) = 100 − R_tot_(%) − T_tot_(%), taking into account that the substrate transmittance and therefore the total transmittance (T_tot_) is zero for the samples studied here.

## 3. Results and Discussion

### 3.1. Structural Characteristics as a Function of the TiO_2_ Film Thickness

**Figure 1:**
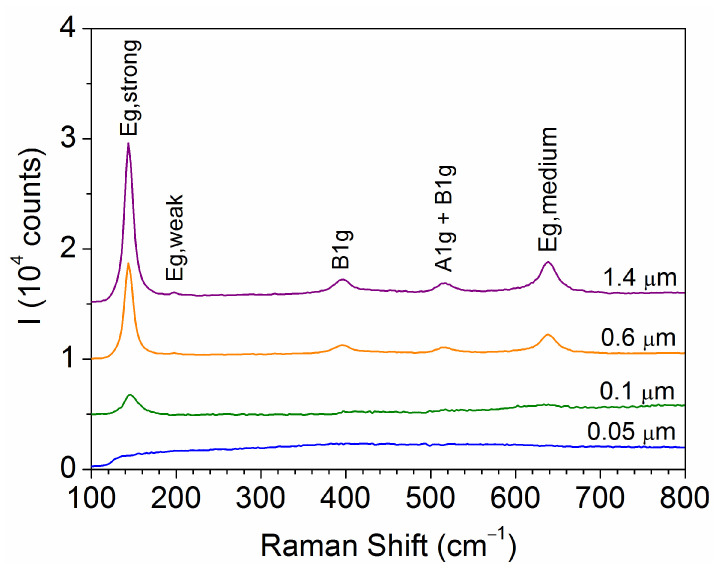
Raman spectra obtained for films with different thicknesses. The characteristic vibrational modes of anatase TiO_2_ are identified.

The influence of the film thickness (t) on the microstructure of the samples is illustrated in Figure 1, where the Raman spectra exhibit vibrational modes characteristics of the anatase TiO_2_ structure [19] in all cases except for the thinnest film (t = 0.05 μm), which shows no vibration signal.

**Figure 2:**
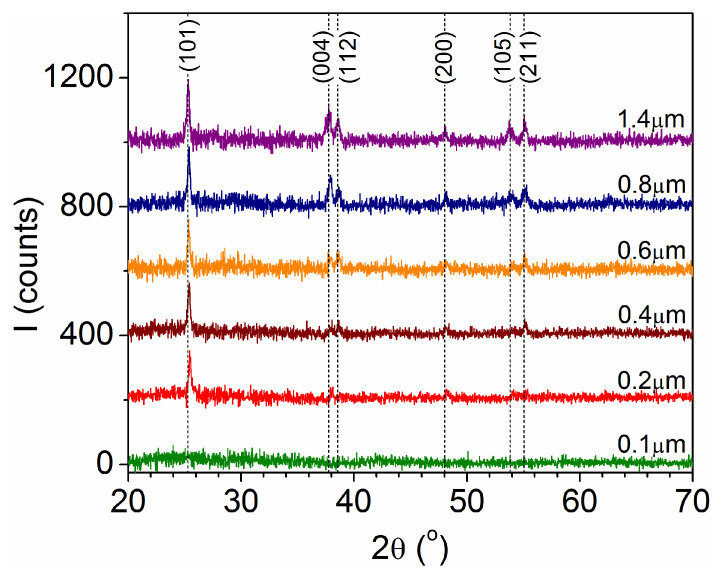
XRD patterns obtained for films with different thicknesses. The vertical dashed lines represent the angular positions corresponding to the standard TiO_2_ anatase (PDF #00-021-1272).

The XRD patterns in Figure 2 exhibit clear diffraction peaks for t ≥ 0.2 μm, corresponding to the (101), (004), (112), (200), (105), and (211) planes of the TiO_2_ anatase phase (card PDF #00-021-1272), without any other reflections that could be ascribed to a different phase. Below a minimal thickness, here, t < 0.2 μm; as for other TiO_2_ films [9,16], it is common for amorphous or crystalline structures with extremely small crystallite sizes lower than 4 nm to be present but not observed by XRD [17]. A more detailed analysis reveals that the angular positions of the peaks (2θ_(hkl)_) are slightly above those given for the standard powdered TiO_2_ anatase, moving to the standard values when the film thickness increases. This indicates the existence of uniform compressive stress in the thinnest films [20], which is often found in coatings made by the condensation of energetic species [21,22], being, in general, weaker for thicker layers [22]. In addition, the intensity of the (004) peak increases with the film thickness, while the intensity of the (101) peak remains practically unchanged. This is interesting because the {101} facets have the lowest formation energy [23], but the {001} facets (set of planes that includes (004)) favor the dissociative adsorption of water and other molecules [24,25], being much more reactive for the photocatalytic degradation of contaminants in liquid and gas phases [7,26].

For the different films represented in Figure 2, the lattice parameters (a, c) and the unit cell volume (V) have been obtained from the measured interplanar spacings corresponding to the main diffraction peaks, d_(101)_ and d_(004)_, using the following equations for the tetragonal lattice structure by applying Bragg’s law [27]:

(1) $$\frac{1}{d_{\left(hkl\right)}^{2}}=\frac{h^{2}+k^{2}}{a^{2}}+\frac{l^{2}}{c^{2}},$$

(2) $$V=a^{2}c$$

Furthermore, the mean crystallite size (D) for the main facets has been obtained from the full width at half maximum (β) of the corresponding diffraction peak and the wavelength (λ) of the X-ray source, according to the Scherrer formula, expressed as follows [17]:

(3) $$D_{\left(hkl\right)}=\frac{0.94\lambda }{\beta cos\theta_{\left(hkl\right)}}$$

**Figure 3:**
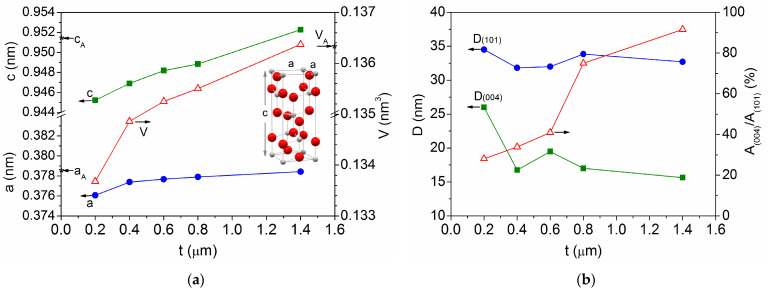
Evolution of the crystalline characteristics with the sputtered film thickness: (**a**) lattice parameters and unit cell volume; and (**b**) mean crystallite size and ratio between the main diffraction peaks. The values corresponding to the standard anatase (a_A_, c_A_, V_A_) are included for comparison.

Figure 3 shows the evolution of these structural features with the film thickness, where the standard powdered anatase values (a_A_ = 0.3785 nm, c_A_ = 0.9514 nm, and V_A_ = 0.1363 nm^3^) are included for comparison (Figure 3a). There is a certain thickness range, 0.4 μm ≤ t ≤ 1.4 μm, where the mean crystallite size remains practically constant, D_(101)_ = 33 ± 1 nm and D_(004)_ = 17 ± 1 nm in Figure 3b, while the lattice parameters and the unit cell volume grow linearly in the ranges a = 0.3774–0.3784 nm, c = 0.9469–0.9523 nm, and V = 0.1349–0.1364 nm^3^. Therefore, the standard anatase values are finally reached in the thickest sample. However, for a lower thickness of t = 0.2 μm, a further decrease in the lattice parameter (a = 0.3761 nm) and the volume (V = 0.1337 nm^3^) is observed, together with a significant increase in the mean crystallites sizes, especially in the (004) plane, with D_(101)_ = 35 ± 1 nm and D_(004)_ = 26 ± 1 nm. Concerning the proportion of the principal planes in the samples, which are represented as the ratio between the respective areas r = A_(004)_/A_(101)_ in Figure 3b, an increase is observed from r = 30–40% for the thinnest samples, above the random value r = 20% for the anatase powder, up to r = 75% for t = 0.8 μm, and r = 90% for t = 1.4 μm. Thus, for the present experimental conditions, the TiO_2_ film growth starts with highly compressed quasi-randomly orientated crystallites that evolve into more relaxed and preferentially (004)-orientated crystallites as the film thickness increases with deposition time.

**Figure 4:**
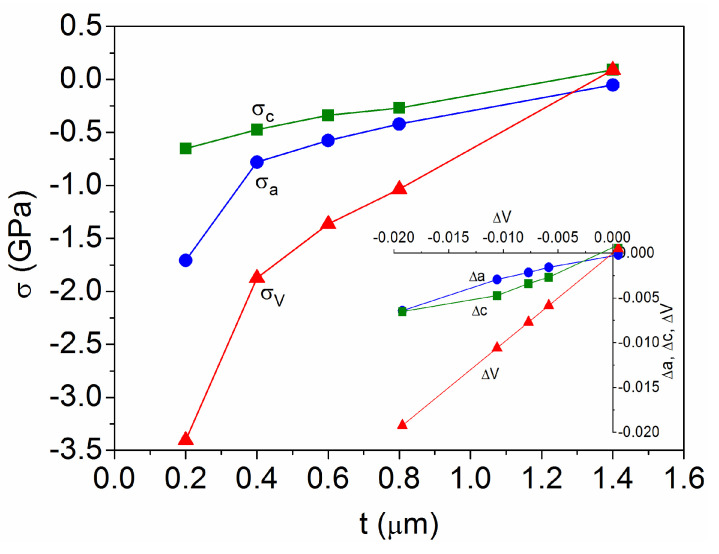
Evolution of the crystalline lattice stress with the sputtered film thickness. The inset shows the interrelation between the respective strain values.

Strain (ε) in the crystalline lattice is given as a fractional change in either length or volume, while stress (σ) is a quantity that describes the magnitude of the forces causing the deformation. At the elastic limit, both parameters are related by the Young modulus or modulus of elasticity Y_i_, along the i-axis, as follows [28]:

(4) $$\sigma_{i}=Y_{i}\varepsilon_{i},$$

where Y_a_ = 266 GPa, Y_c_ = 100 GPa, and the bulk modulus is B = 177 GPa for the anatase TiO_2_ [29,30]. This relationship results in the stress values depicted in Figure 4 for the samples with different thicknesses, taking the respective strain as the displacement of the experimental data with respect to the standard anatase value as follows [31]:

(5a) $$\sigma_{a}(GPa)=Y_{a}\Delta a=266(a-a_{A})/a_{A}$$

(5b) $$\sigma_{c}(GPa)=Y_{c}\Delta c=100(c-c_{A})/c_{A}$$

(5c) $$\sigma_{V}(GPa)=B∆V=177(V-V_{A})/V_{A}$$

Therefore, positive values of the strain and stress indicate that the crystals are in tensile mode while negative values are due to compressive deformation [32]. Positive stress in the range of 0.2–2.0 GPa is observed for anatase TiO_2_ powders [28,33], without a direct dependence on the crystallite size from 10 to 80 nm [33]. Otherwise, negative stress ranging from −0.7 to −12 GPa has been reported for anatase TiO_2_ thin films [34,35], where the lattice distortion rate shows a strong size dependence when the crystallite size is less than 4 nm, remaining unaffected by sizes above 8 nm [34]. Figure 4 shows that, here, the compressive stress decreases as the film thickness increases, from σ_V_ = −3.5 GPa for t = 0.2 μm to σ_V_ = 0.0 GPa for t = 1.4 μm, regardless of the crystallite size, which is always larger than 15 nm (Figure 3b). For each sputtered TiO_2_ film, the compressive stress along the c-axis is lower than for the a-axis, due to the different values of the respective Young modulus, but the strain contribution is slightly higher for the c-axis, as represented by the inset in Figure 4. In this regard, it is known that [001] is the soft direction in the anatase phase, giving the c-direction good compressibility [29]. Furthermore, it should be noted that the following relationship is observed: ∆V = 2∆a + ∆c ~ 3Δa.

Several studies have been performed to understand the residual stress in sputtered thin films [22,32,36]. Under high kinetic energy conditions, compressive strain predominates because the bombarded ions hitting the growing surface cause local atomic displacements leading to the incorporation of incoming atoms into the deposited film, resulting in a smaller than typical volume [22]. On the other hand, the grain coalescence mechanism, which occurs when a film is grown by any synthesis technique, involves a tensile strain [32]. Viewing the lattice distortion as a result of stress mechanisms of a different sign, the evolution observed for the present TiO_2_ samples can also be related to a weakening of the overall stress when the (004) texture increases with the film thickness. As in other works, where the stress reduction is associated with a relaxation of the material to the phase or crystalline preferred orientation that minimizes the excess energy in the growing film [21,22].

### 3.2. Optical Characteristics as a Function of the TiO_2_ Film Thickness

**Figure 5:**
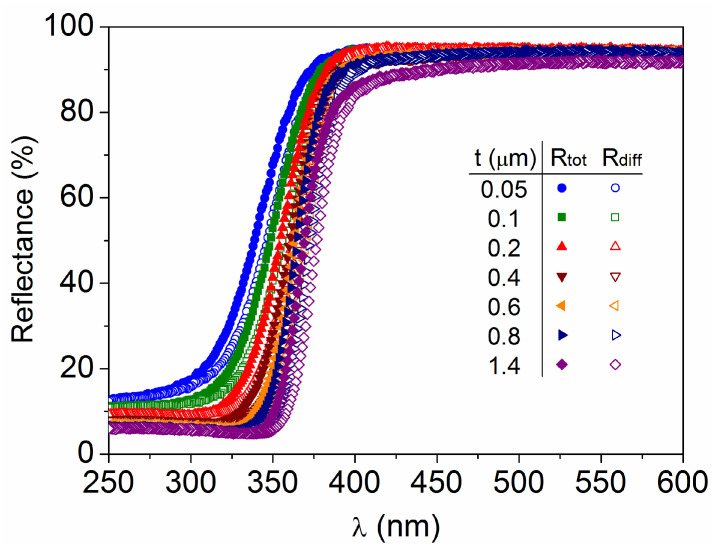
Total and diffuse reflectance spectra as a function of the light wavelength for the TiO_2_ films with different thicknesses.

The optical reflectance spectra obtained for the different TiO_2_ films are represented in Figure 5, where it can be seen that, for each sample, the total reflectance is slightly higher than the diffuse reflectance (R_tot_ > R_diff_) in the transition region from low to high reflectance values. This transition is due to the optical absorption of photons with energy (E = hc/λ) greater than that of the semiconductor band gap (E ≥ E_g_). Thus, reflectance or absorptance spectra can be used to determine E_g_, but there is some debate about the most appropriate method to determine its value [15,37]. Analysis of direct reflectance (R_dir_ = R_tot_ − R_diff_) will also be performed later in this subsection. Regarding the increase in diffuse reflectance in thinner layers, it could be related to the increase in defect density but not to surface roughness because the latter tends to decrease for thinner films [13].

**Figure 6:**
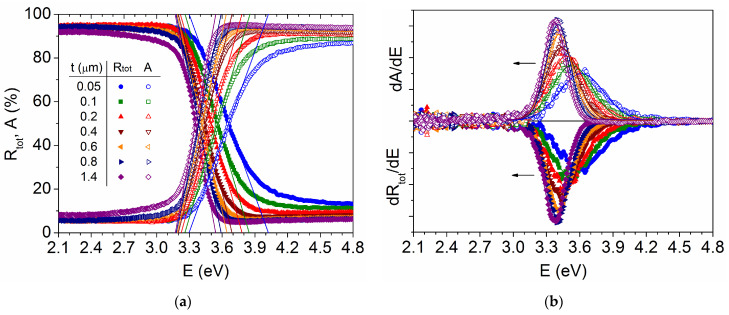
Calculation of the band gap energy from the total reflectance and absorptance spectra of TiO_2_ films with different thicknesses: (**a**) application of the tangent method, and (**b**) derivative method.

The band gap energy has been calculated from the linear fit of the absorption edge (also called the tangent method), by extrapolation to R_tot_ = 100% (or A = 0%) [37], as illustrated in Figure 6a. The value thus obtained changes from E_g_ = 3.30 eV for t = 0.05 μm to E_g_ = 3.17 eV for t = 1.4 μm, which is lower than that reported for analogous TiO_2_ films [38]. This is because the extrapolation is performed here to determine the energy at which absorption starts, while other methods focus on the high-absorption region. It should be noted that in the tangent method the extrapolation of absorptance is always performed towards A = 0%, but in the case of reflectance, it is sometimes directed towards R_tot_ = 0%, which is not really correct and gives large discrepancies in the values obtained for the same sample [37]. In fact, Figure 6a shows that the extrapolation to R_tot_ = 0% gives much higher energies in the range of 4.02–3.54 eV. On the other hand, in amorphous or polycrystalline materials, the onset of absorption may be due to defect states located at energies lower than the band-to-band transition [39]. Looking for a compromise solution between both extremes, the point at which R_tot_ = A = 50% has been determined for the different samples, which varies from E_g_ = 3.66 eV for t = 0.05 μm to E_g_ = 3.36 eV for t = 1.4 μm. These latter values are very close to those obtained by the derivative method (dR_tot_/dE or dA/dE) [15], which are found in the range of 3.61–3.37 eV, as shown in Figure 6b. The symmetry of the derivative peaks indicates the presence of a single phase in the samples, anatase detected by XRD, while the transition region becomes narrow as the film thickness increases.

**Figure 7:**
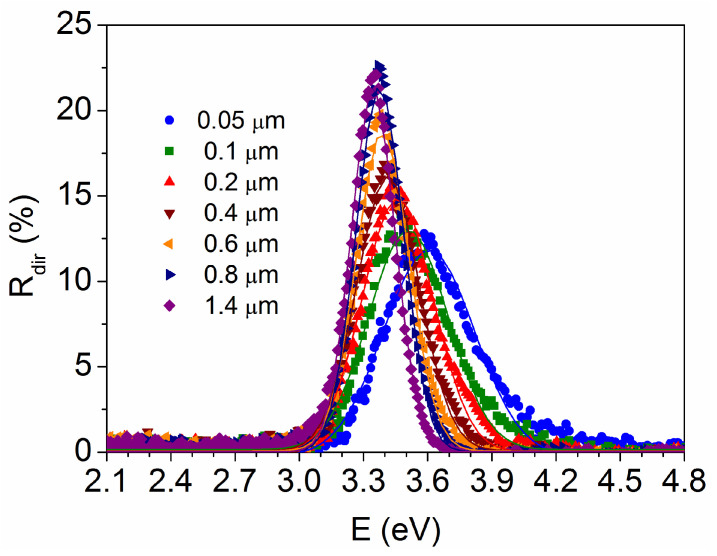
Calculation of the band gap energy from the direct reflectance spectra of TiO_2_ films with different thicknesses.

In order to eliminate the diffuse contribution, which is not only related to the film thickness, the analysis of the direct reflectance is shown in Figure 7. Diffuse reflectance refers to radiation that is reflected in all directions, which is highly dependent on the surface roughness [40], while direct or specular reflectance has a defined reflection angle with respect to the incident radiation and occurs at an optically smooth surface. Here, the direct component shows a peak that behaves like the derivative dA/dE, but it is located at somewhat lower values, changing from E_g_ = 3.58 eV for t = 0.05 μm to E_g_ = 3.34 eV for t = 1.4 μm. This is because the diffuse reflectance contribution increases in the high-absorption region, just above the direct reflectance maximum, causing a slight shift in the absorption edge in R_tot_ (or A) towards higher energies.

**Figure 8:**
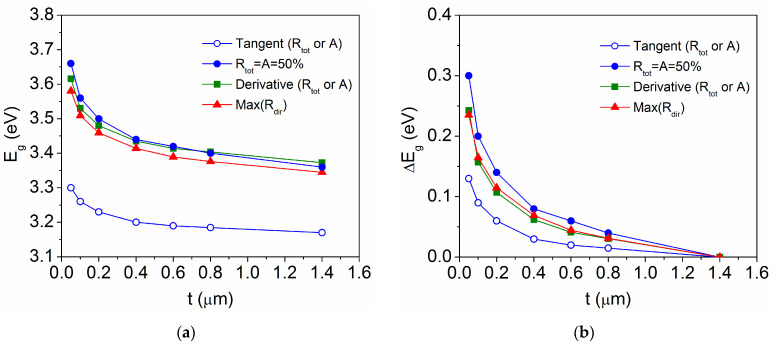
Evolution of (**a**) the band gap energy E_g_ and (**b**) the variation ΔE_g_, determined by several methods for TiO_2_ films with different thickness.

A comparison of the E_g_ values obtained by the different methods is represented in Figure 8 as a function of TiO_2_ film thickness. For each method, the variation ∆E_g_ is calculated as the difference between the value for the stressed film (σ ≠ 0 when t < 1.4 μm) and that for the stress-free coating (σ = 0 for t = 1.4 μm) as follows:

(6) $$\Delta E_{g}=E_{g}(\sigma \neq 0)-E_{g}\left(\sigma =0\right)$$

The accurate determination of E_g_ and ∆E_g_ is essential for the quantitative analysis of the observed bandgap energy widening. The tangent method (focused on the region where optical absorption begins) is found to underestimate both E_g_ and ∆E_g_ for the various samples, while the other three methods (analyzing the high-absorption region) yield similar values. Data obtained from the direct reflectance spectra can be considered more reliable since they are only affected by the film thickness, being close to the values obtained by the derivative method. It is interesting to note that R_dir_ cannot always be used because it is zero for TiO_2_ powder samples, for which R_tot_ = R_diff_, but it is well defined for the present layers. Both R_dir_ and dR_tot_/dE result in a maximum ∆E_g_ = 0.24 eV for the thinnest film with t = 0.05 μm, for which the equality method (R_tot_ = A = 50%) gives a slightly higher ∆E_g_ = 0.30 eV. Relative changes of the same order have been reported for other semiconductor thin films when the crystallite size decreases [41] or the lattice strain increases [42,43]. Moreover, band gap energies above 3.5 eV are found for amorphous TiO_2_ films [44], in good agreement with the absence of diffraction peaks for t ≤ 0.1 μm (Figure 2).

### 3.3. Relationship Between Band Gap Energy and Unit Cell Volume in TiO_2_ Thin Films

**Figure 9:**
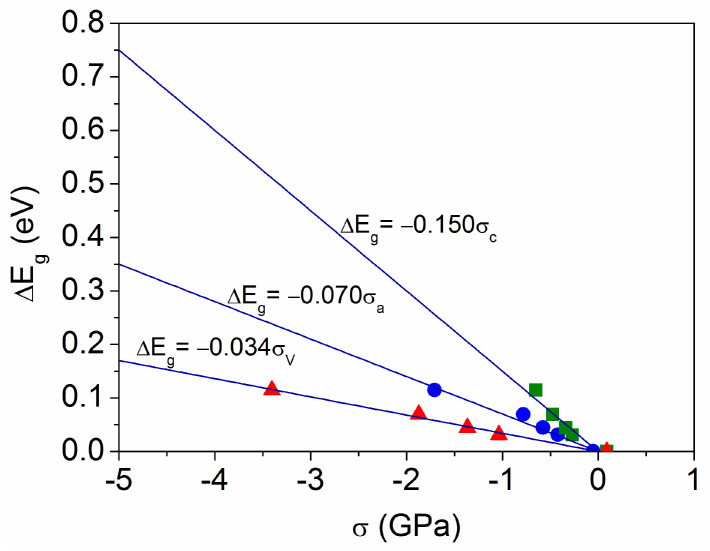
Variation in the band gap energy as a function of stress (σ_a_ circles, σ_c_ squares, and σ_V_ triangles) in the anatase crystalline lattice.

Figure 9 shows the variation in the band gap energy (∆E_g_ corresponding to Max(R_dir_) in Figure 8b) as a function of the respective stress values (σ_a_, σ_c_, and σ_V_ depicted in Figure 4). Although larger variations are reached in Figure 8b (∆E_g_ = 0.16 eV for t = 0.1 μm and ∆E_g_ = 0.24 eV for t = 0.05 μm), no strain data were obtained for these films because they did not show diffraction peaks. Nevertheless, an extrapolation of the fits in Figure 9 allows us to estimate the stress values that would result in a larger ∆E_g_. In addition, the size-dependent lattice distortion model predicts a dependence Δa∝−0.025/D(nm) [34], resulting in ∆a ~ −0.013 for a crystallite size D = 2 nm, which is too low to generate observable diffraction peaks. This corresponds to σ_a_ ~ −3.46 GPa and thus to ΔE_g_ ~ 0.24 eV according to the extrapolation performed in Figure 9, which is consistent with the band gap widening obtained for the sample with t = 0.05 μm.

In deformation potential theory [45], the band gap change under stress is expressed by the following:

(7) $$\Delta E_{g}=b_{a}\sigma_{a}+b_{c}\sigma_{c},$$

where b_a_ and b_c_ are the band gap pressure coefficients for epitaxial and uniaxial stress, respectively. Epitaxial (or biaxial) deformation is considered due to lattice mismatch or thermal strain during film growth, whereas uniaxial deformation could result from the application of an external uniaxial pressure. In the case of hydrostatic pressure (σ_c_ = σ_a_), the expression can be simplified to ∆E_g_ = (b_a_ + b_c_) σ_a_, where (b_a_ + b_c_) < 0 for the anatase TiO_2_ structure [29]. However, this does not apply for the present samples, since Figure 4 shows that σ_c_ ~ 0.5σ_a_ and therefore ∆E_g_ ~ (b_a_ + 0.5b_c_) σ_a_. This results in (b_a_ + 0.5b_c_) = −0.07 eV/GPa, according to the fit in Figure 9.

An analogous expression based on the volume stress is proposed here as follows:

(8) $$\Delta E_{g}=b_{V}\sigma_{V},$$

which gives a band gap pressure coefficient b_V_ = −0.034 eV/GPa according to the corresponding data in Figure 9. This value is close to that obtained using first-principles band structure calculations for the anatase TiO_2_ lattice with only uniaxial stress [29] or epitaxial strain [46].

**Figure 10:**
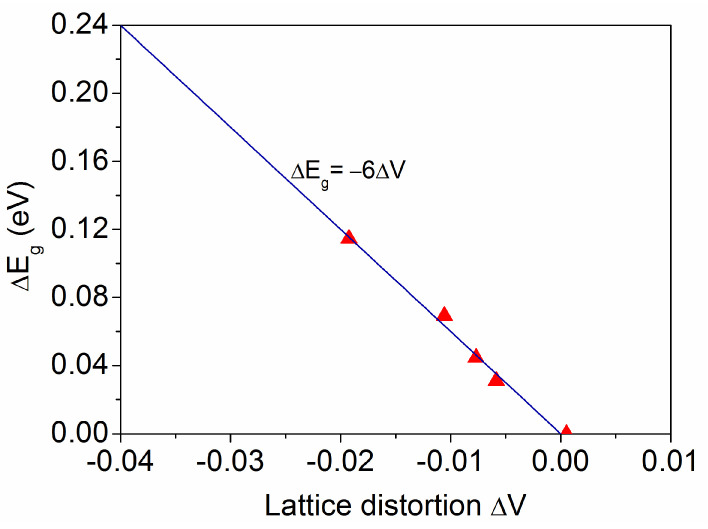
Variation in the band gap energy as a function of the volume strain in the anatase crystalline lattice.

By relating the variation in band gap energy to the strain instead of the stress, an expression is obtained that directly relates the optical features to the crystalline lattice distortion as follows:

(9) $$\Delta E_{g}=b_{V}^{*}\Delta V,$$

with a new band gap pressure coefficient $b_{V}^{*}$ = −6 eV, obtained from the linear fit represented in Figure 10, which is related to the previous b_V_ by the corresponding bulk modulus of elasticity, expressed as follows:

(10) $$b_{V}^{*}=b_{V}B,$$

where B = 177 GPa for anatase TiO_2_ [30].

The extrapolation performed in Figure 10 allows us to consider that the largest gap widening obtained in Section 3.2, ∆E_g_ = 0.24 eV for t = 0.05 μm, is consistent with a small crystallite size D ~ 2 nm. This value is too low to be observable by XRD and may generate a lattice distortion ∆a ~ −0.025/D(nm) = −0.013, resulting in ΔV = 3Δa = −0.039. Furthermore, the density functional theory predicts a higher band gap energy for amorphous TiO_2_ than for anatase, with a maximum value of ∆E_g_ = 0.32 eV [47] that is supported by the experimental data [44].

## 4. Conclusions

TiO_2_ thin films prepared by sputtering on unheated fiber glass wafers have shown clear diffraction peaks corresponding to the tetragonal anatase phase when the film thickness is 0.2 μm ≤ t ≤ 1.4 μm, with increasing lattice parameters and unit cell volume in the ranges a = 0.3761–0.3784 nm, c = 0.9452–0.9523 nm, and V = 0.1337–0.1364 nm^3^ as the thickness increases. The mean crystallite sizes are D_(101)_ ≥ 33 nm and D_(004)_ ≥ 17 nm for the various samples. Thus, the film growth starts with compressed quasi-randomly orientated crystallites (volume stress σ_V_ = −3.5 GPa for t = 0.2 μm) that evolve into relaxed and preferentially (004) orientated crystallites, reaching the standard anatase values at t ~ 1.4 μm with σ_V_ = 0.0 GPa. For each coating, the compressive stress along the c-axis is lower than for the a-axis (σ_c_ < σ_a_), due to the different values of the respective Young modulus, but the strain contribution is slightly higher for the c-axis (∆c ≥ ∆a) with respect to the following relation: ∆V = 2∆a + ∆c ~ 3Δa. Below a minimum thickness (t < 0.2 μm), the films have an amorphous nature or small crystallite sizes less than 4 nm, which are not observable by X-ray diffraction.

Since the accurate determination of the band gap energy is essential for its quantitative analysis, a detailed comparison has been made between the E_g_ value obtained by different methods from the measured total and diffuse reflectance spectra (R_tot_, R_diff_) and from the corresponding direct reflectance (R_dir_ = R_tot_ − R_diff_) and absorptance (here, A = 100 − R_tot_ for opaque substrates). For each method, the variation ∆E_g_ has been calculated as the difference between the value for the stressed film (σ ≠ 0 when t < 1.4 μm) and that for the stress-free coating (σ = 0 for t = 1.4 μm). A priori, the values obtained from R_dir_ are considered more reliable, as they are only affected by the film thickness, and are confirmed by their coincidence with those obtained by the derivative method (dR_tot_/dE or dA/dE). Both result in a maximum ∆E_g_ = 0.24 eV for the thinnest film, with E_g_ ~ 3.60 eV for t = 0.05 μm, while the equality method (R_tot_ = A = 50%) gives a slightly higher ∆E_g_ = 0.30 eV. Otherwise, the tangent method (extrapolation to R_tot_ = 100% or A = 0%) underestimates both E_g_ and ∆E_g_ for the various samples. Finally, the change in band gap energy has been directly related to the crystalline lattice distortion by the relation ∆E_g_ (eV) = −6∆V, for TiO_2_ film thicknesses 0.2 μm ≤ t ≤ 1.4 μm. From this expression, it can be considered that a larger gap widening, such as ∆E_g_ = 0.24 eV for t = 0.05 μm, would correspond to a small crystallite size D ~ 2 nm, which is too low to be observable by X-ray diffraction and may generate a lattice distortion ∆a ~ −0.025/D(nm) = −0.013, resulting in ΔV ~ 3Δa = −0.039.

The deformation of the anatase crystal lattice can modify the optoelectronic properties of TiO_2_ thin films in a significant and predictable way. Therefore, a method is presented that allows tuning the band gap energy in order to optimize photon harvesting from the light source and improve the performance of photovoltaic and photocatalytic devices based on this material.

## Data Availability

The original contributions presented in the study are included in the article; further inquiries can be directed to the author.
